# Supplementary material for: Chemoton 2.0: Autonomous Exploration of Chemical Reaction Networks
Source: J Chem Theory Comput. 2022 Aug 4;18(9):5393–409. doi: 10.1021/acs.jctc.2c00193 (PMC11516015; doi:10.1021/acs.jctc.2c00193)
Supplement: Supplementary file 1 — ct2c00193_si_001.pdf [file ct2c00193_si_001.pdf]

Chemoton 2.0: Exploration of Chemical Reaction Networks

Jan P. Unsleber[1], Stephanie A. Grimmel[2], and Markus Reiher[3]

Laboratory for Physical Chemistry, ETH Zurich,
Vladimir-Prelog-Weg 2, 8093 Zurich, Switzerland

Supporting Information

[1]ORCID: 0000-0003-3465-5788

[2]ORCID: 0000-0001-7633-6123

[3]Corresponding author; e-mail: markus.reiher@phys.chem.ethz.ch; ORCID: 0000-0002-9508-1565

S1 Test Reactions

Table S1: Reference reactions that we probed for in the test runs.

	Reaction	Ref.
**1.1**	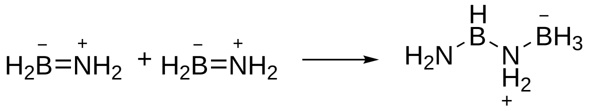	1, 2
**1.2**	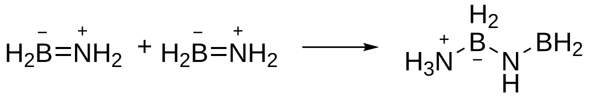	1, 2
**1.3**	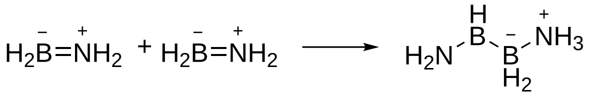	1, 2
**2.1**	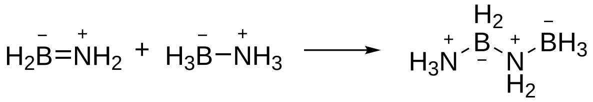	1, 2
**2.2**	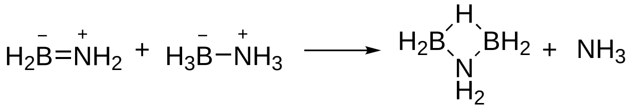	1, 2
**2.3**	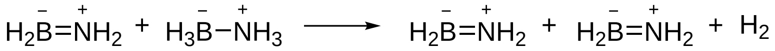	1, 2
**2.4**	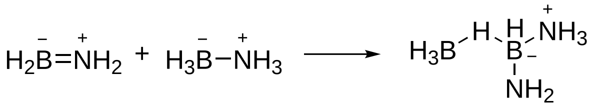	1, 2
**2.5**	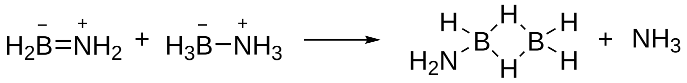	1, 2
**3.1**	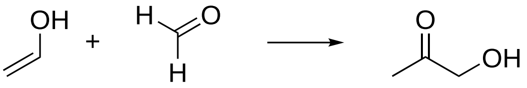	1, 2
**3.2**	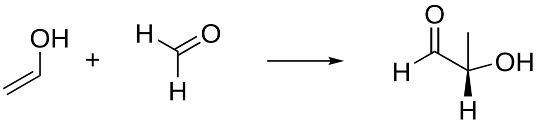	1, 2
**3.3**	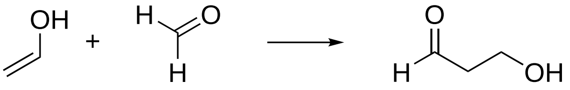	1, 2
**3.4**	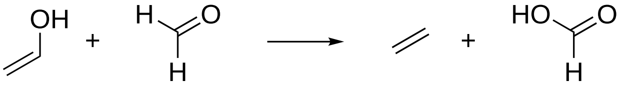	1, 2
**3.5**	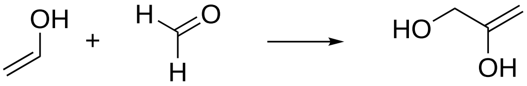	1, 2
**3.6**	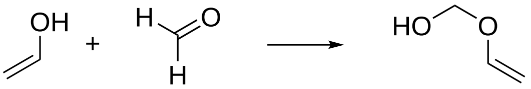	1, 2
**3.7**	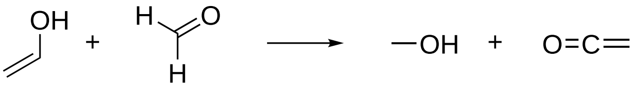	1, 2

**3.8**	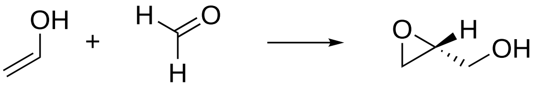	*1*, *2*
**3.9**	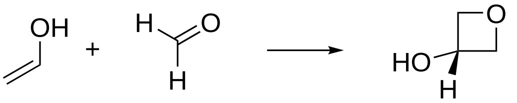	*1*, *2*
**4.1**	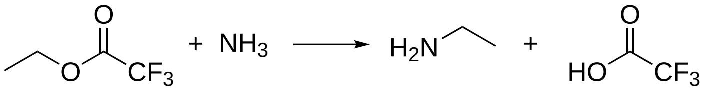	*1*, *2*
**4.2**	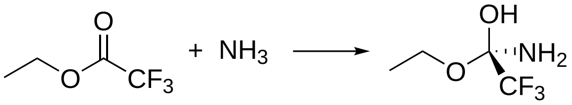	*1*, *2*
**5.1**	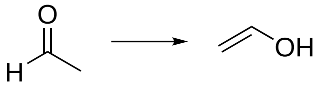	*1*, *2*
**6.1**	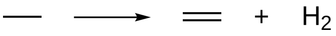	*1*, *2*
**7.1**	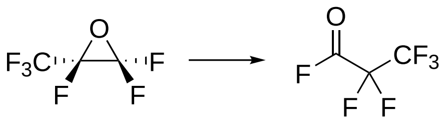	*1*, *2*
**7.2**	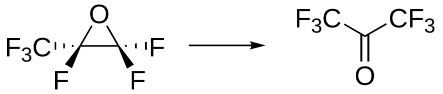	*1*, *2*
**8.1**	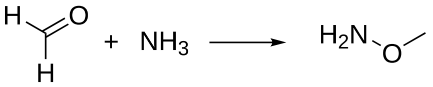	*1*, *2*
**8.2**	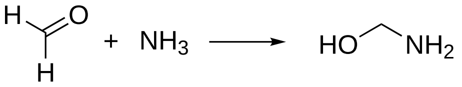	*1*, *2*
**9.1**	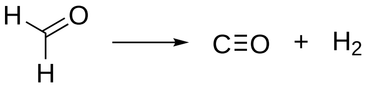	*1*, *2*
**10.1**	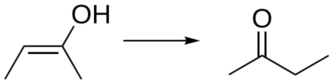	*1*, *2*
**10.2**	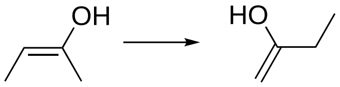	*1*, *2*
**10.3**	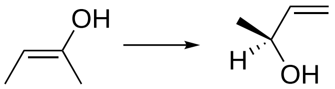	*1*, *2*
**10.4**	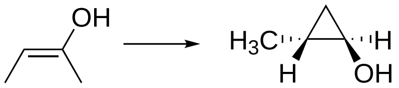	*1*, *2*
**10.5**	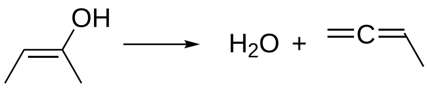	*1*, *2*
**10.6**	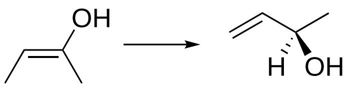	*1*, *2*
**11.1**	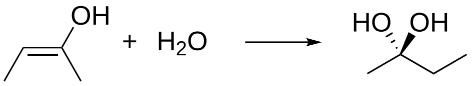	*1*, *2*

11.2 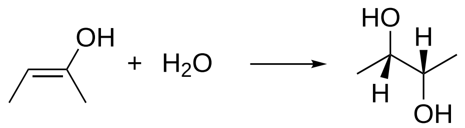

1, 2

11.3 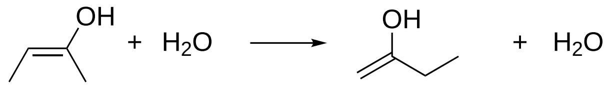

1, 2

11.4 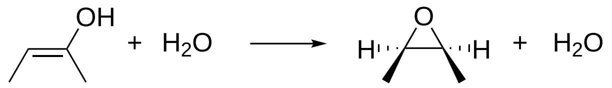

1, 2

11.5 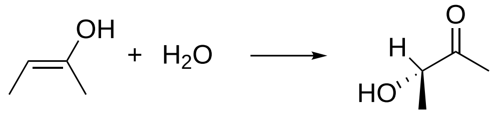

1, 2

12.1 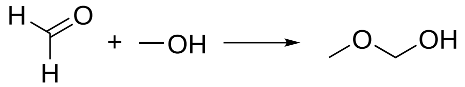

1, 2

12.2 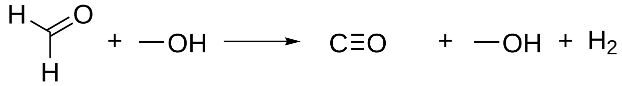

1, 2

12.3 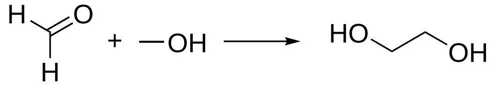

1, 2

13.1 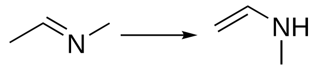

1, 2

13.2 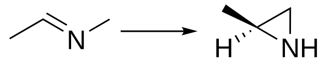

1, 2

13.3 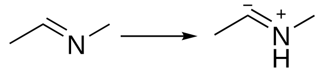

1, 2

13.4 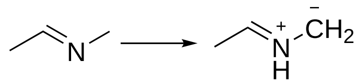

1, 2

13.5 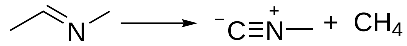

1, 2

14.1 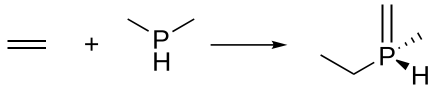

1, 2

15.1 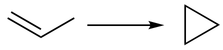
1, 2

16.1 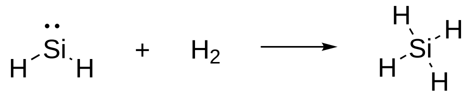

1, 2

17.1 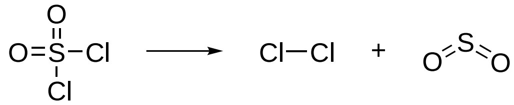

1, 2

18.1 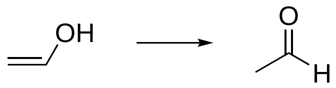

1, 2

19.1 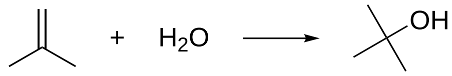

1, 2

20.1 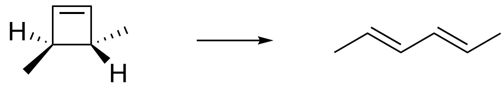

1, 2

21.1 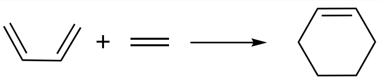 *1, 2*

21.2 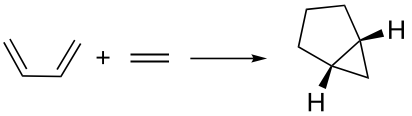 *1, 2*

21.3 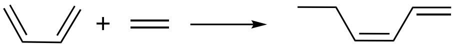 *1, 2*

21.4 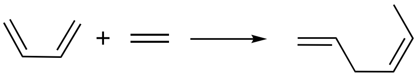 *1, 2*

21.5 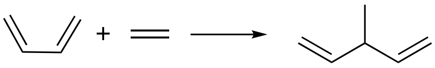 *1, 2*

21.6 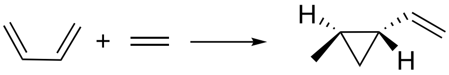 *1, 2*

21.7 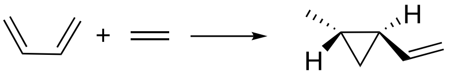 *1, 2*

21.8 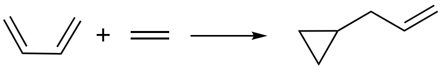 *1, 2*

21.9 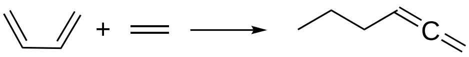 *1, 2*

21.10 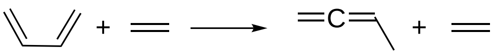 *1, 2*

22.1 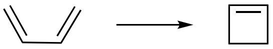 *1, 2*

23.1 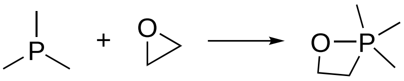 *1, 2*

24.1 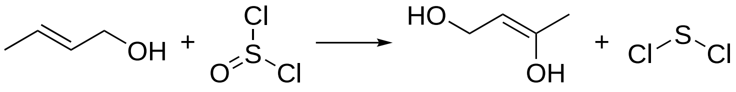 *1, 2*

24.2 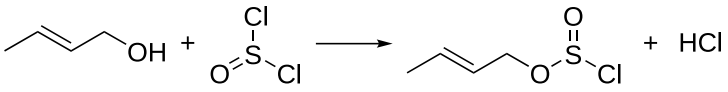 *1, 2*

24.3 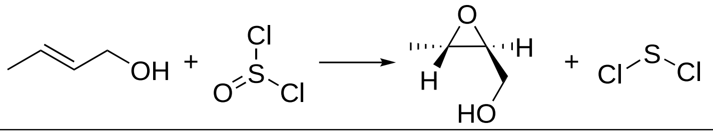 *1, 2*

25.1 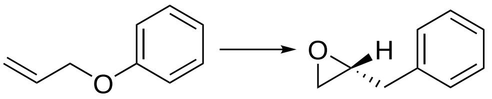 *1, 2*

25.2 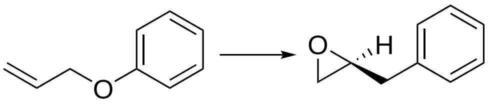 *1, 2*

25.3 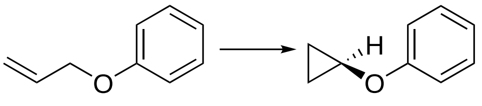 *1, 2*

25.4 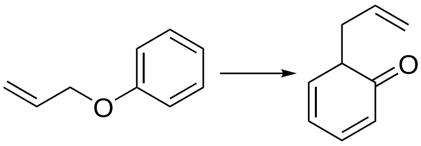 *3*

**26.1**	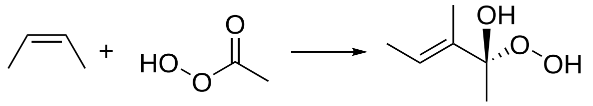	*1, 2*
**26.2**	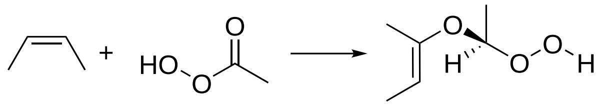	*1, 2*
**26.3**	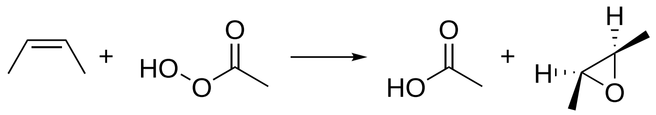	*1, 2*
**27.1**	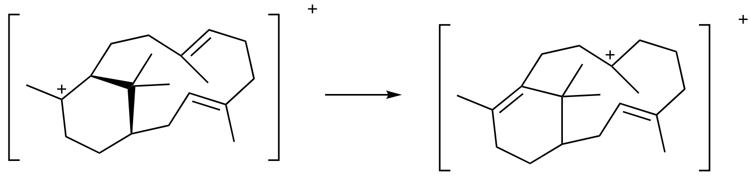	*1, 2*
**27.2**	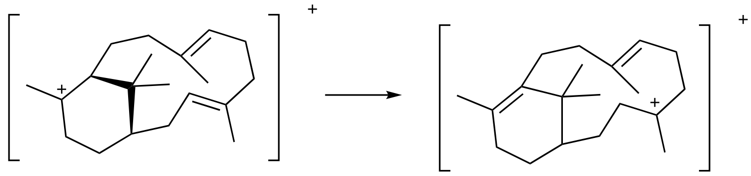	*1, 2*
**28.1**	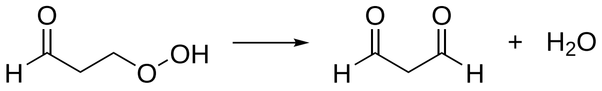	*4, 5*
**28.2**	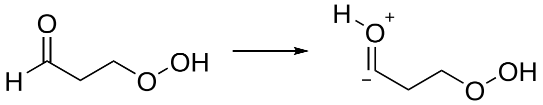	*4, 5*
**28.3**	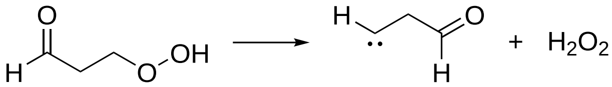	*4, 5*
**28.4**	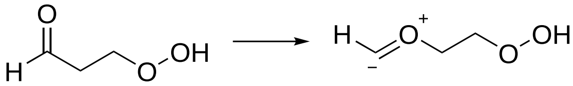	*4, 5*
**28.5**	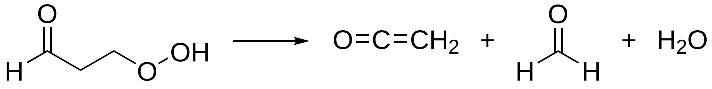	*4, 5*
**28.6**	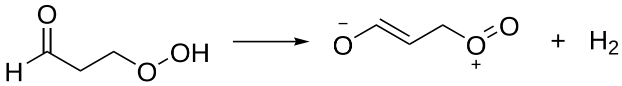	*4, 5*
**28.7**	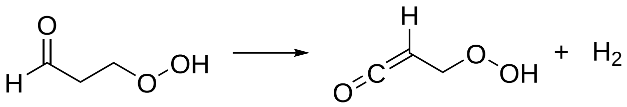	*4, 5*
**28.8**	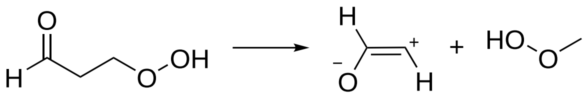	*4, 5*
**28.9**	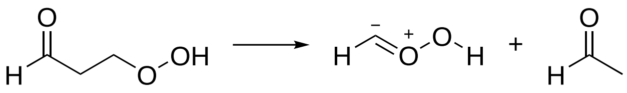	*4, 5*
**28.10**	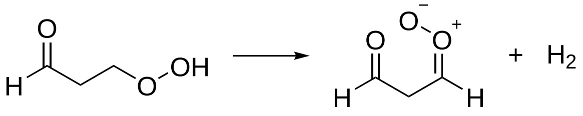	*4, 5*

28.11 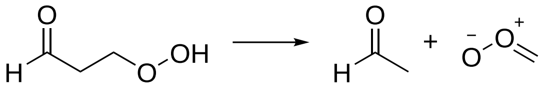 *4, 5*

28.12 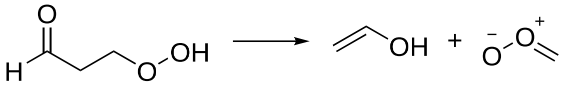 *4, 5*

28.13 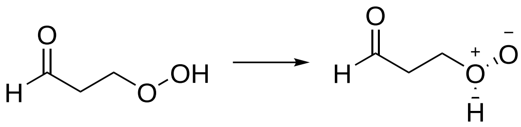 *4, 5*

28.14 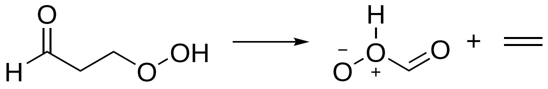 *4, 5*

28.15 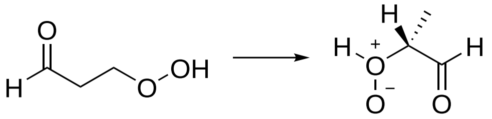 *4, 5*

28.16 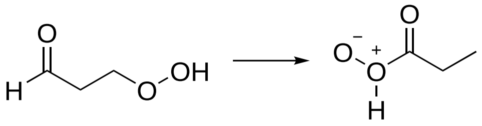 *4, 5*

28.17 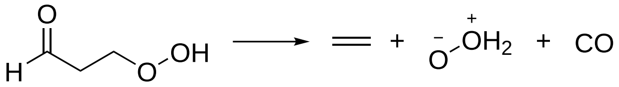 *4, 5*

28.18 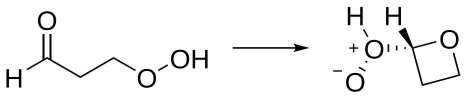 *4, 5*

28.19 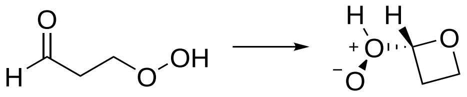 *4, 5*

28.20 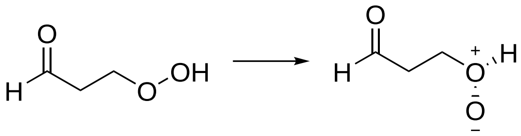 *4, 5*

28.21 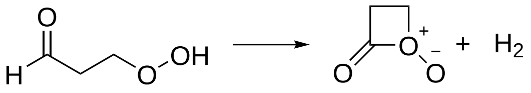 *4, 5*

28.22 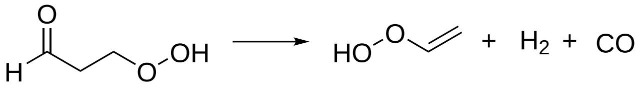 *4, 5*

28.23 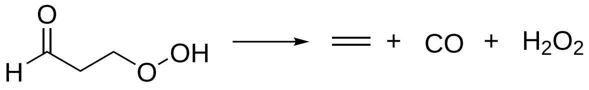 *4, 5*

28.24 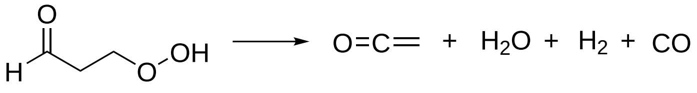 *4, 5*

28.25 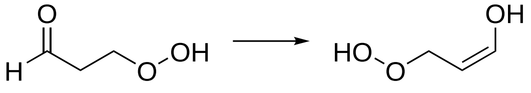 *4, 5*

28.26 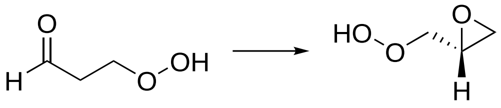 *4, 5*

28.27 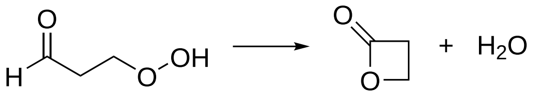 *4, 5*

28.28 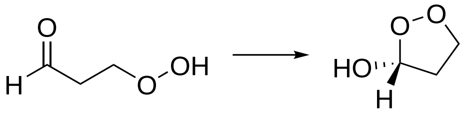 *4, 5*

28.29 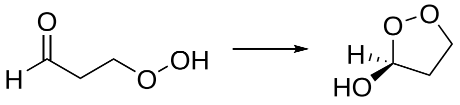 *4, 5*

28.30 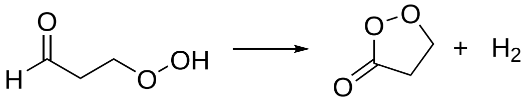 *4, 5*

28.31 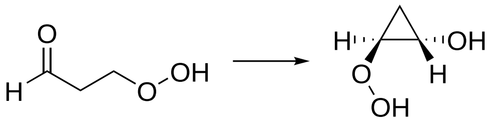 *4, 5*

28.32 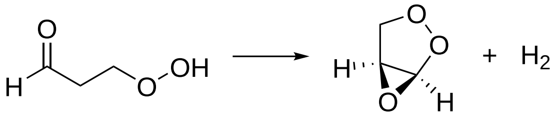 *4, 5*

28.33 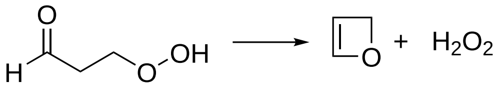 *4, 5*

28.34 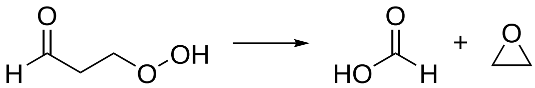 *4, 5*

28.35 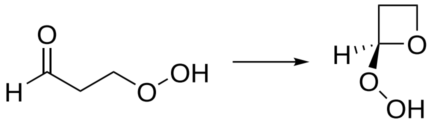 *4, 5*

28.36 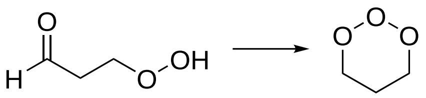 *4, 5*

28.37 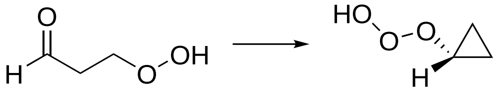 *4, 5*

28.38 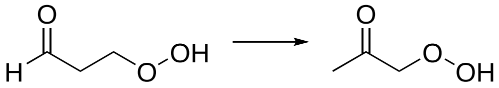 *4, 5*

28.39 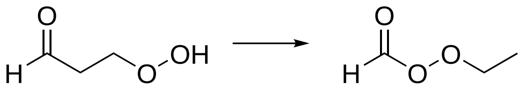 *4, 5*

28.40 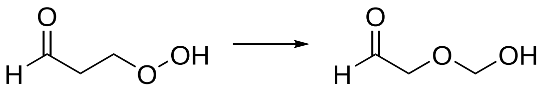 *4, 5*

28.41 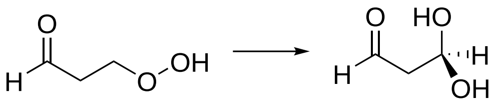 *4, 5*

28.42 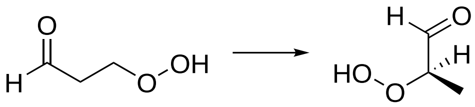 *4, 5*

**28.43**	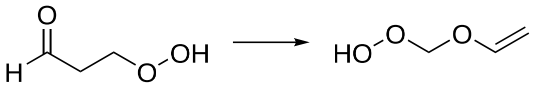	*4, 5*
**28.44**	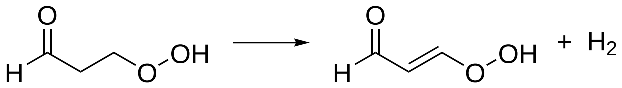	*4, 5*
**28.45**	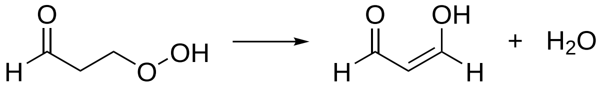	*4, 5*
**28.46**	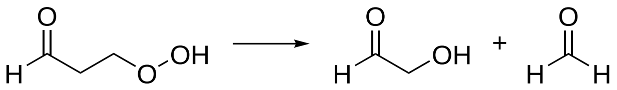	*4, 5*
**28.47**	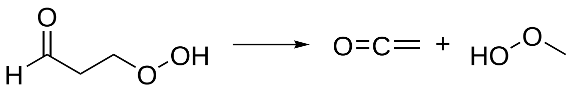	*4, 5*
**28.48**	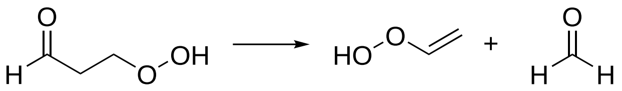	*4, 5*
**28.49**	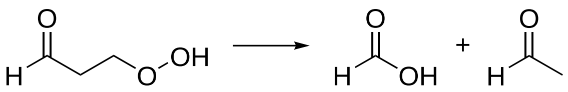	*4, 5*
**28.50**	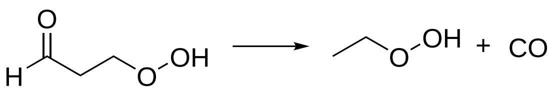	*4, 5*
**28.51**	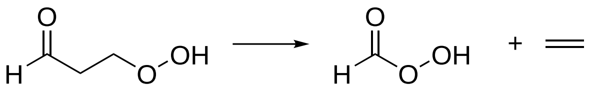	*4, 5*
**28.52**	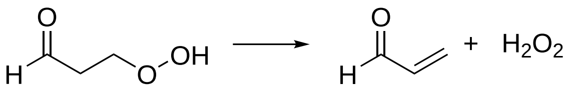	*4, 5*
**28.53**	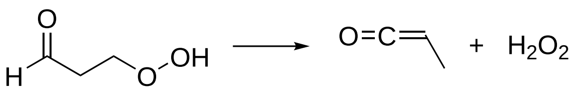	*4, 5*
**28.54**	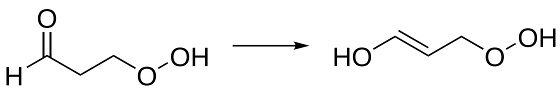	*4, 5*
**29.1**	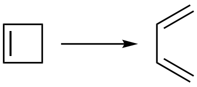	*6*
**30.1**	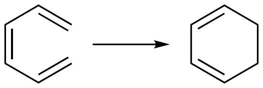	*6*
**31.1**	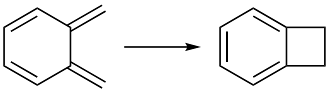	*6*
**32.1**	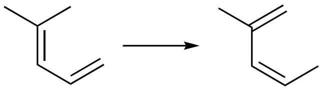	*6*
**33.1**	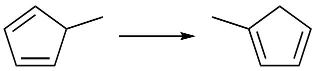	*6*
**34.1**	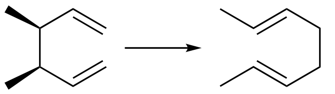	*6*

**35.1**	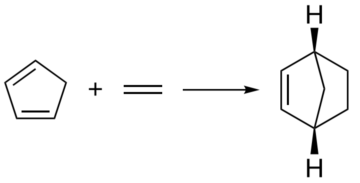	*6*
**36.1**	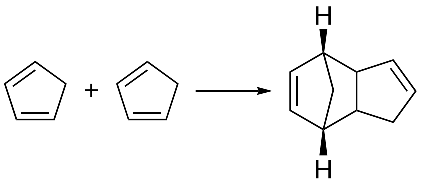	*6*
**37.1**	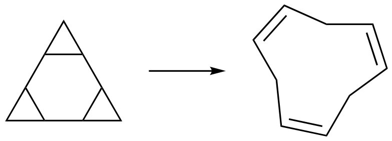	*6*
**38.1**	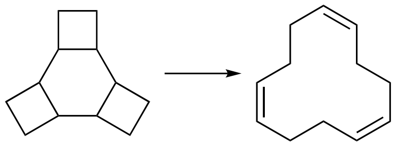	*6*
**39.1**	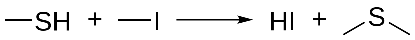	*3*
**39.2**	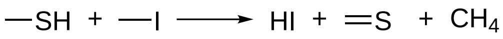	*3*
**39.3**	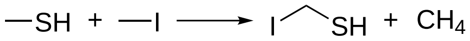	*3*
**40.1**	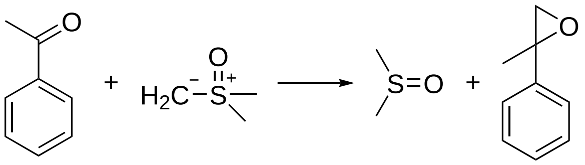	*3*
**40.2**	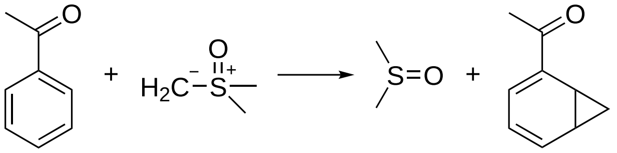	*3*
**40.3**	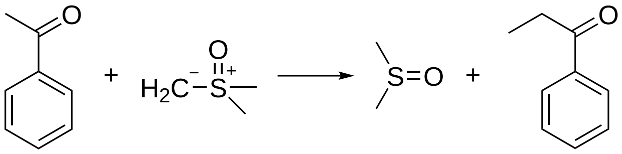	*3*
**40.4**	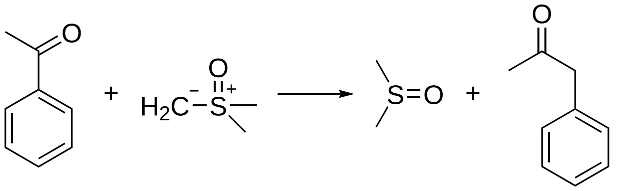	*3*
**41.1**	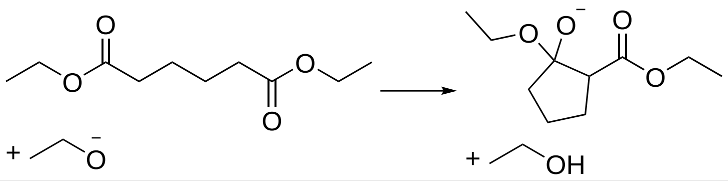	*3*

**42.1**	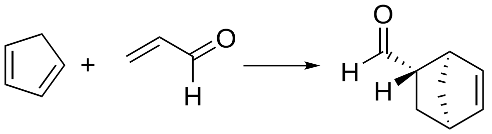	*3*
**42.2**	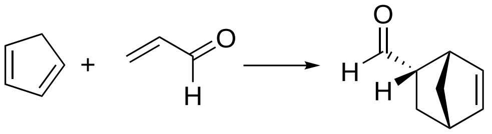	*3*
**42.3**	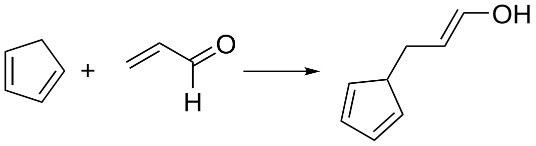	*3*
**42.4**	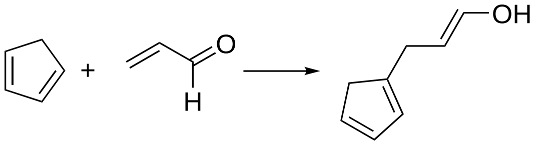	*3*
**42.5**	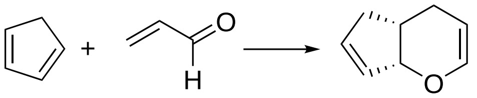	*3*
**43.1**	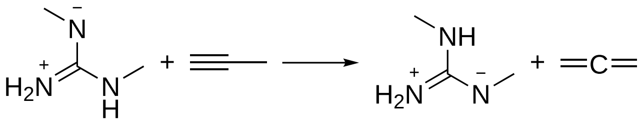	*3*
**43.2**	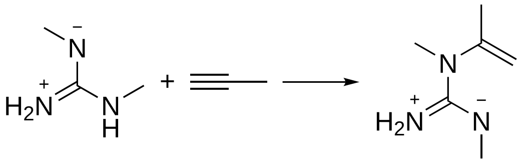	*3*
**44.1**	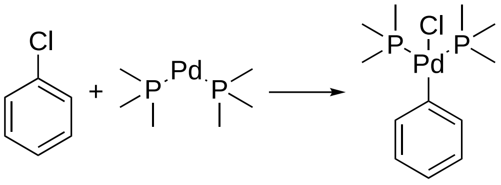	*3*
**45.1**	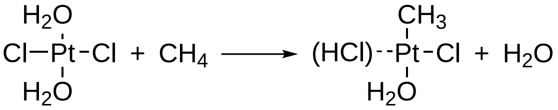	*7, 8*
**46.1**	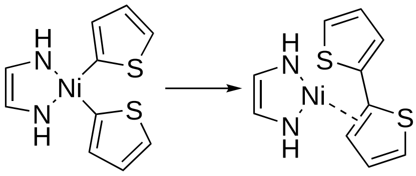	*7, 9*
**47.1**	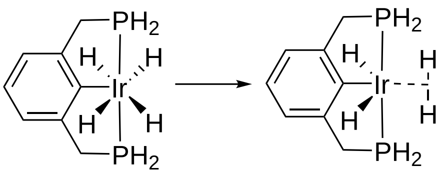	*7*

**48.1**	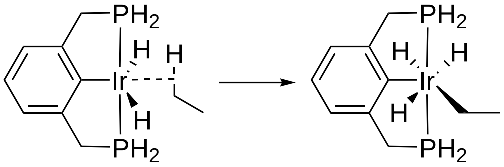	*7, 10*
**49.1**	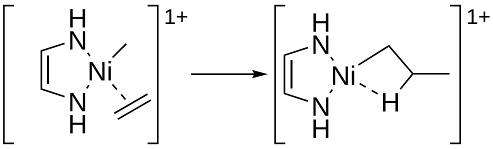	*7*
**50.1**	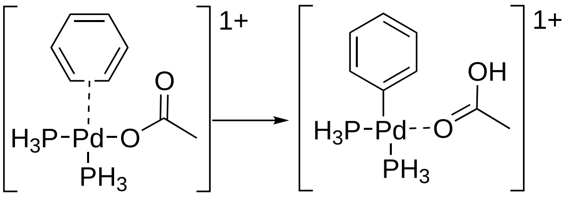	*7*
**51.1**	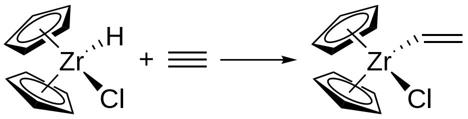	*11*
**52.1**	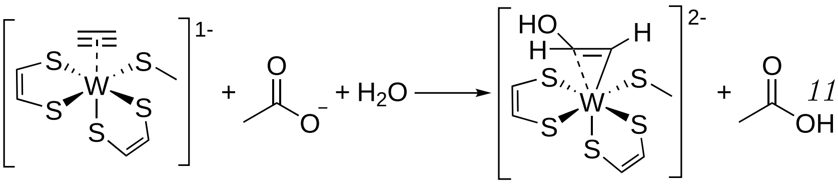	*11*
**53.1**	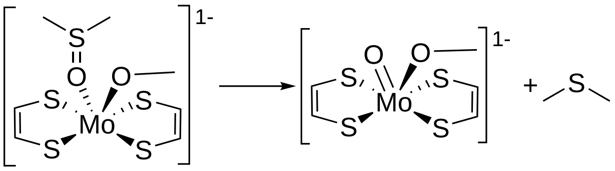	*11*
**54.1**	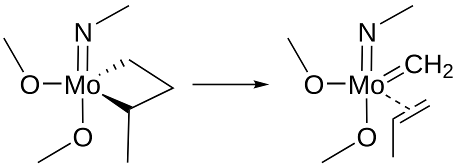	*11*
**55.1**	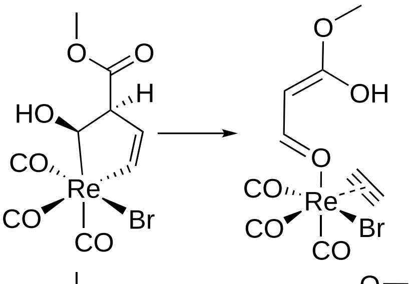	*11*
**55.2**	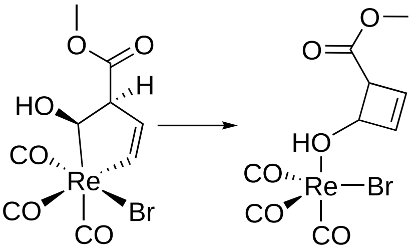	*11*

**55.3**	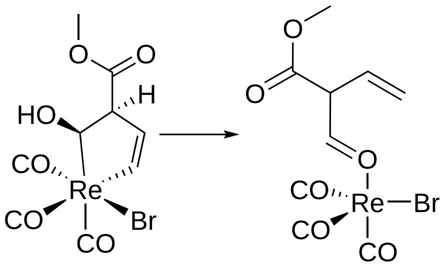	*11*
**56.1**	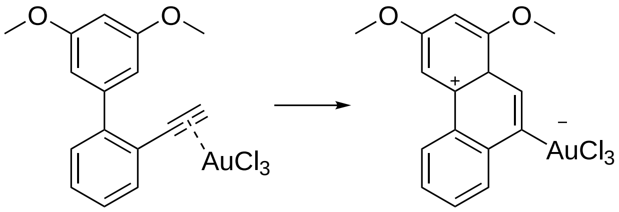	*11*
**57.1**	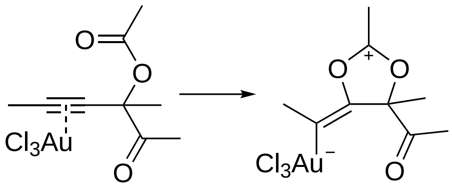	*11*
**58.1**	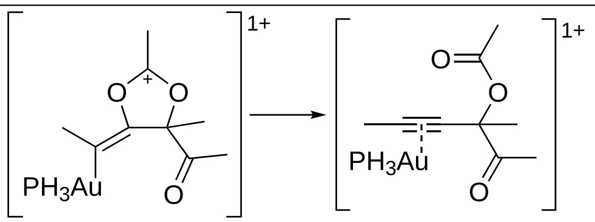	*11*
**59.1**	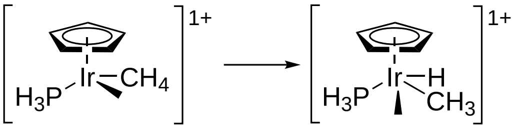	*11*
**60.1**	$H-\overset{+}{N}\equiv\overset{-}{C} \longrightarrow H-C\equiv N$	*12*
**61.1**	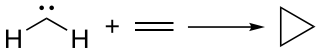	*13*
**62.1**	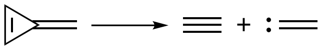	*13*
**63.1**	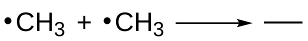	*14*
**64.1**	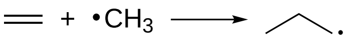	*12*
**65.1**	$H\cdot + \cdot OH \longrightarrow H_2 + \ddot{O}$	*12*
**66.1**	$\cdot NH_2 + CH_4 \longrightarrow NH_3 + \cdot CH_3$	*12*
**67.1**	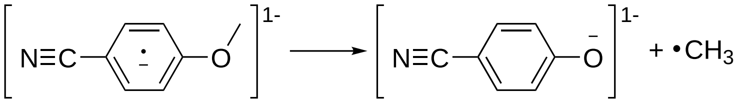	*15*
**68.1**	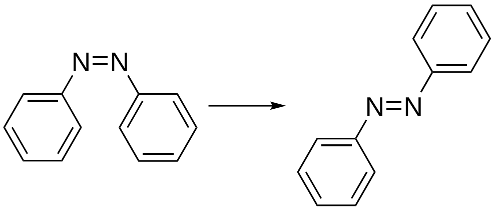	*16*

**69.1**	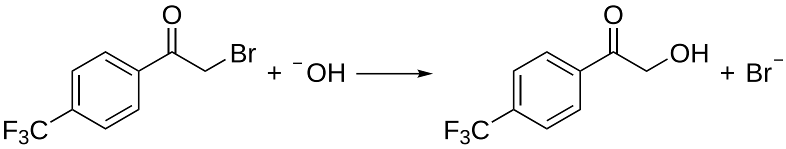	*17*, *18*
**69.2**	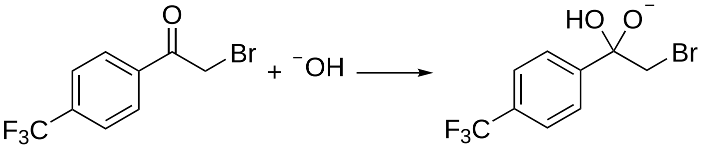	*17*, *18*

S2 Detailed Exploration Settings

The molecular connectivity used for graph interpretation was determined from interatomic distances: Two atoms were considered to be bound if their distance had been below the sum of their covalent radii plus 0.4 Å. MOLASSEMBLER's [*19*, *20*] bond stereopermutators were instantiated on bonds that were detected based on the distance criterion and moreover had a Mayer bond order [*21*, *22*] of more than 1.4. Additional settings used for structure optimizations of reference structures and elementary step trials are given in Table S2.

Table S2: Settings employed during structure optimizations and elementary step trial calculations. For further explanations consider the manuals and source codes of SCINE READUCT[*23*, *24*] and PUFFIN.[*25*]

Calculation Type	Setting	Value
Structure Optimization	`max_scf_iterations`	1000
	`convergence_max_iterations`	1000
	`convergence_step_max_coefficient`	2.0e-3
	`convergence_step_rms`	1.0e-3
	`convergence_gradient_max_coefficient`	2.0e-4
	`convergence_gradient_rms`	1.0e-4
	`convergence_delta_value`	1.0e-6
	`convergence_requirement`	3
	`bfgs_use_trust_radius`	`True`
	`bfgs_trust_radius`	0.2
	`geoopt_coordinate_system`	`cartesianWithoutRotTrans`
Elementary Step Trial Calculation		
	`max_scf_iterations`	1000
NT Scan	`convergence_max_iterations`	600
	`nt_total_force_norm`	0.1
	`sd_factor`	1.0
	`nt_use_micro_cycles`	`True`
	`nt_fixed_number_of_micro_cycles`	`True`
	`nt_number_of_micro_cycles`	10
	`nt_filter_passes`	10

Transition State Optimization	convergence_max_iterations	1000
	convergence_step_max_coefficient	2.0e-3
	convergence_step_rms	1.0e-3
	convergence_gradient_max_coefficient	2.0e-4
	convergence_gradient_rms	1.0e-4
	convergence_requirement	3
	convergence_delta_value	1e-6
	optimizer	Bofill
	bofill_trust_radius	0.2
	geoopt_coordinate_system	cartesianWithoutRotTrans
IRC	convergence_max_iterations	100
	sd_factor	0.2
	sd_use_trust_radius	True
	sd_trust_radius	0.05
	sd_dynamic_multiplier	1.2
	irc_initial_step_size	0.3
	stop_on_error	False
	convergence_step_max_coefficient	2.0e-3
	convergence_step_rms	1.0e-3
	convergence_gradient_max_coefficient	2.0e-4
	convergence_gradient_rms	1.0e-4
	convergence_delta_value	1.0e-6
	irc_coordinate_system	cartesianWithoutRotTrans
IRC Endpoint Optimization	convergence_max_iterations	1000
	convergence_step_max_coefficient	2.0e-3
	convergence_step_rms	1.0e-3
	convergence_gradient_max_coefficient	2.0e-4
	convergence_gradient_rms	1.0e-4
	convergence_requirement	3
	convergence_delta_value	1e-6
	bfgs_use_trust_radius	True
	bfgs_trust_radius	0.2
	geoopt_coordinate_system	cartesianWithoutRotTrans
Product Optimization	convergence_max_iterations	1000
	convergence_step_max_coefficient	2.0e-3
	convergence_step_rms	1.0e-3
	convergence_gradient_max_coefficient	2.0e-4
	convergence_gradient_rms	1.0e-4
	convergence_requirement	3
	convergence_delta_value	1e-6
	bfgs_use_trust_radius	True
	bfgs_trust_radius	0.4
	geoopt_coordinate_system	cartesianWithoutRotTrans

S3 Detailed Results

Table S3: Found and missed reference reactions using different quantum chemical methods and algorithms in the elementary step search of CHEMOTON. Orange highlighting indicates that the structure optimization of the reference reactants or products was not successful, *e.g.*, resulting in the structure to dissociate into different molecules. Note that failures may be a consequence of the approximate structure model.

	GFN2		DFTB3
	NT1	NT2	NT2
**1.1**	✓	✓	–
**1.2**	✓	✓	–
**1.3**	✓	✓	–
**2.1**	✓	✓	–
**2.2**	✓	✓	–
**2.3**	✓	✓	–
**2.4**	×	×	–
**2.5**	×	×	–
**3.1**	✓	×	×
**3.2**	✓	✓	×
**3.3**	✓	✓	✓
**3.4**	✓	✓	✓
**3.5**	×	✓	✓
**3.6**	✓	✓	✓
**3.7**	✓	✓	✓
**3.8**	×	×	×
**3.9**	✓	✓	✓
**4.1**	✓	✓	–
**4.2**	✓	✓	–
**5.1**	✓	✓	✓
**6.1**	✓	✓	✓
**7.1**	✓	✓	–
**7.2**	✓	✓	–
**8.1**	✓	×	×
**8.2**	✓	✓	✓
**9.1**	✓	✓	✓
**10.1**	✓	✓	✓
**10.2**	✓	✓	✓
**10.3**	✓	✓	✓
**10.4**	✓	✓	✓
**10.5**	✓	✓	✓
**10.6**	✓	✓	✓
**11.1**	✓	✓	×
**11.2**	✓	✓	×
**11.3**	×	✓	✓
**11.4**	×	×	×
**11.5**	×	×	×
**12.1**	✓	✓	✓
**12.2**	✓	✓	✓
**12.3**	✓	✓	✓
**13.1**	✓	✓	✓
**13.2**	✓	✓	×
**13.3**	✓	✓	✓
**13.4**	✓	✓	✓
**13.5**	✓	✓	✓
**14.1**	✓	✓	✓
**15.1**	✓	✓	✓
**16.1**	✓	✓	–
**17.1**	✓	✓	–
**18.1**	✓	✓	✓
**19.1**	✓	✓	✓
**20.1**	✓	✓	✓
**21.1**	✓	✓	✓
**21.2**	✓	✓	×
**21.3**	✓	✓	✓
**21.4**	✓	✓	✓
**21.5**	✓	✓	✓
**21.6**	×	×	×

	GFN2		DFTB3
	NT1	NT2	NT2
**21.7**	✓	✓	×
**21.8**	✓	✓	×
**21.9**	✓	✓	✓
**21.10**	✓	✓	✓
**22.1**	✓	✓	✓
**23.1**	×	✓	×
**24.1**	✓	×	–
**24.2**	✓	✓	–
**24.3**	✓	✓	–
**25.1**	×	✓	✓
**25.2**	×	✓	✓
**25.3**	✓	✓	✓
**25.4**	✓	✓	✓
**26.1**	✓	✓	✓
**26.2**	×	×	✓
**26.3**	✓	✓	✓
**27.1**	✓	✓	✓
**27.2**	✓	✓	✓
**28.1**	✓	✓	✓
**28.2**	✓	✓	✓
**28.3**	×	×	×
**28.4**	✓	✓	✓
**28.5**	✓	✓	✓
**28.6**	✓	✓	✓
**28.7**	✓	✓	✓
**28.8**	✓	✓	×
**28.9**	✓	✓	×
**28.10**	×	✓	✓
**28.11**	✓	✓	✓
**28.12**	×	✓	✓
**28.13**	✓	✓	✓
**28.14**	✓	✓	✓
**28.15**	✓	✓	×
**28.16**	×	✓	×
**28.17**	×	×	×
**28.18**	×	×	×
**28.19**	×	×	×

	GFN2		DFTB3
	NT1	NT2	NT2
**28.20**	×	✓	✓
**28.21**	×	✓	✓
**28.22**	×	✓	✓
**28.23**	✓	✓	✓
**28.24**	×	×	✓
**28.25**	✓	✓	✓
**28.26**	×	✓	✓
**28.27**	✓	✓	×
**28.28**	✓	✓	✓
**28.29**	✓	✓	✓
**28.30**	✓	✓	✓
**28.31**	✓	✓	×
**28.32**	×	×	×
**28.33**	×	✓	✓
**28.34**	✓	✓	✓
**28.35**	×	×	✓
**28.36**	×	×	×
**28.37**	✓	✓	×
**28.38**	✓	✓	✓
**28.39**	✓	✓	✓
**28.40**	×	✓	✓
**28.41**	×	✓	✓
**28.42**	✓	✓	✓
**28.43**	×	✓	✓
**28.44**	✓	✓	✓
**28.45**	×	✓	✓
**28.46**	✓	✓	✓
**28.47**	✓	✓	✓
**28.48**	✓	✓	✓
**28.49**	✓	✓	×
**28.50**	✓	✓	✓
**28.51**	✓	✓	✓
**28.52**	×	✓	✓
**28.53**	×	✓	×
**28.54**	✓	✓	✓
**29.1**	✓	✓	✓
**30.1**	✓	✓	✓

	GFN2		DFTB3
	NT1	NT2	NT2
**31.1**	✓	✓	✓
**32.1**	✓	✓	✓
**33.1**	✓	✓	✓
**34.1**	✓	✓	✓
**35.1**	✓	✓	✓
**36.1**	✓	✓	✓
**37.1**	✓	✓	✓
**38.1**	×	✓	✓
**39.1**	✓	✓	–
**39.2**	✓	✓	–
**39.3**	✓	✓	–
**40.1**	✓	✓	✓
**40.2**	✓	✓	✓
**40.3**	✓	✓	✓
**40.4**	✓	✓	✓
**41.1**	×	×	×
**42.1**	✓	✓	✓
**42.2**	✓	✓	✓
**42.3**	×	✓	✓
**42.4**	×	×	×
**42.5**	×	✓	✓
**43.1**	✓	✓	✓
**43.2**	×	✓	✓
**44.1**	×	×	–
**45.1**	×	×	–
**46.1**	×	×	–
**47.1**	×	×	–
**48.1**	×	✓	–
**49.1**	×	✓	–
**50.1**	×	×	–
**51.1**	✓	✓	–
**52.1**	×	×	–
**53.1**	×	✓	–
**54.1**	×	×	–
**55.1**	×	×	–
**55.2**	✓	✓	–
**55.3**	×	×	–

	GFN2		DFTB3
	NT1	NT2	NT2
**56.1**	×	×	–
**57.1**	×	×	–
**58.1**	✓	✓	–
**59.1**	✓	✓	–
**60.1**	×	×	×
**61.1**	×	×	×
**62.1**	×	✓	×
**63.1**	×	×	×
**64.1**	×	✓	×
**65.1**	×	×	×
**66.1**	✓	✓	×
**67.1**	×	✓	✓
**68.1**	×	✓	✓
**69.1**	×	×	–
**69.2**	×	×	–
$\sum(\checkmark)$	120	147	106

Table S4: Total numbers of reactions found, elementary steps found and elementary step calculations during GFN2-based calculations. The elementary steps are not deduplicated. In the first column, the number of reactions used as reference is given. Note that many of the references given did not aim to list all possible reactions.

	# Reactions			# Elementary Steps		# Elementary Step Trials (Success rate/%)			
#	Ref.	NT1	NT2	NT1	NT2	NT1		NT2	
**1**	3	6	9	140	97	530	(26.4)	530	(18.3)
**2**	5	12	15	222	179	1048	(21.2)	1048	(17.1)
**3**	9	45	41	129	97	390	(33.1)	390	(24.9)
**4**	2	24	31	231	224	1596	(14.5)	1596	(14.0)
**5**	1	3	7	19	226	102	(18.6)	846	(26.7)
**6**	1	1	2	3	452	193	(1.6)	1855	(24.4)
**7**	2	14	27	111	983	498	(22.3)	6940	(14.2)
**8**	2	5	7	52	39	136	(38.2)	136	(28.7)
**9**	1	2	2	4	8	9	(44.4)	27	(29.6)
**10**	6	30	75	334	6465	1782	(18.7)	28755	(22.5)
**11**	5	15	24	65	129	780	(8.3)	780	(16.5)
**12**	3	14	18	85	65	300	(28.3)	300	(21.7)
**13**	5	14	21	239	2614	831	(28.8)	11395	(22.9)
**14**	1	31	34	416	315	1830	(22.7)	1830	(17.2)
**15**	1	6	11	35	662	324	(10.8)	3662	(18.1)
**16**	1	1	1	7	5	21	(33.3)	21	(23.8)
**17**	1	3	4	14	46	25	(56.0)	109	(42.2)
**18**	1	5	11	17	163	96	(17.7)	846	(19.3)
**19**	1	9	8	59	42	657	(9.0)	657	(6.4)
**20**	1	35	76	733	17161	4458	(16.4)	92836	(18.5)
**21**	10	28	27	435	280	1830	(23.8)	1830	(15.3)
**22**	1	9	22	121	1124	519	(23.3)	6669	(16.9)
**23**	1	38	51	701	757	4186	(16.7)	4186	(18.1)
**24**	3	112	108	368	254	1378	(26.7)	1378	(18.4)
**25**	4	222	826	1643	33395	12142	(13.5)	305255	(10.9)
**26**	3	126	137	846	1010	5886	(14.4)	5886	(17.2)
**27**	2	89	585	187	6809	1378	(13.6)	72874	(9.3)
**28**	54	54	158	225	4552	1228	(18.3)	18491	(24.6)
**29**	1	9	16	172	1767	489	(35.2)	6940	(25.5)
**30**	1	34	96	474	5518	2474	(19.2)	43147	(12.8)
**31**	1	47	187	1111	9727	4396	(25.3)	92836	(10.5)
**32**	1	45	103	911	15577	4584	(19.9)	89055	(17.5)
**33**	1	40	103	581	5955	2391	(24.3)	45059	(13.2)
**34**	1	194	616	1469	73115	18997	(7.7)	487431	(15.0)
**35**	1	58	53	705	387	2211	(31.9)	2211	(17.5)

#	# Reactions Ref.	NT1	NT2	# Elementary Steps NT1	NT2	# Elementary Step Trials (Success Rate/%) NT1		NT2	
**36**	1	190	127	1964	1326	7381	(26.6)	7381	(18.0)
**37**	1	56	203	1924	73178	14406	(13.4)	434799	(16.8)
**38**	1	29	155	91	3229	435	(20.9)	13701	(23.6)
**39**	3	19	15	137	76	465	(29.5)	465	(16.3)
**40**	4	500	501	4547	3646	23855	(19.1)	23855	(15.3)
**41**	1	13	24	62	128	32896	(0.2)	32896	(0.4)
**42**	5	285	236	1040	701	3916	(26.6)	3916	(17.9)
**43**	2	183	184	598	528	5516	(10.8)	5516	(9.6)
**44**	1	56	42	267	199	49326	(0.5)	49326	(0.4)
**45**	1	7	8	388	117	1010	(38.4)	1010	(11.6)
**46**	1	13	62	14	234	300	(4.7)	7671	(3.1)
**47**	1	0	2	0	2	325	(0.0)	8641	(0.0)
**48**	1	30	147	85	1669	496	(17.1)	16204	(10.3)
**49**	1	83	477	1324	21856	9496	(13.9)	241016	(9.1)
**50**	1	31	75	107	1339	378	(28.3)	10499	(12.8)
**51**	1	112	108	445	671	4182	(10.6)	4182	(16.0)
**52**	1	76	126	349	322	14145	(2.5)	14145	(2.3)
**53**	1	14	39	92	1152	378	(24.3)	10499	(11.0)
**54**	1	53	211	85	1787	378	(22.5)	10499	(17.0)
**55**	3	57	302	3959	28254	32510	(12.2)	1017251	(2.8)
**56**	1	64	316	88	1931	630	(14.0)	22571	(8.6)
**57**	1	17	53	25	463	378	(6.6)	10178	(4.5)
**58**	1	38	180	59	1598	406	(14.5)	11339	(14.1)
**59**	1	23	100	48	1507	276	(17.4)	7220	(20.9)
**60**	1	0	0	0	0	3	(0.0)	5	(0.0)
**61**	1	0	0	0	0	171	(0.0)	171	(0.0)
**62**	1	7	12	28	269	161	(17.4)	1898	(14.2)
**63**	1	0	1	0	3	136	(0.0)	136	(2.2)
**64**	1	1	2	20	17	300	(6.7)	300	(5.7)
**65**	1	0	0	0	0	3	(0.0)	3	(0.0)
**66**	1	1	2	18	8	120	(15.0)	120	(6.7)
**67**	1	78	331	860	17674	5815	(14.8)	128537	(13.8)
**68**	1	27	106	54	552	276	(19.6)	6551	(8.4)
**69**	2	0	0	0	0	812	(0.0)	812	(0.0)
$\sum$	184	3443	7659	31542	354635	290976	(10.8)	3441120	(10.3)

Table S5: Comparison of the total number of reactions found, elementary steps found and elementary step calculations carried out during the calculations employing GFN2 and DFTB3, both with the NT2 algorithm. The elementary steps are not deduplicated. In the first column the number of reactions used as reference is given. Note that many of the references given did not aim to list all possible reactions.

	# Reactions			# Elementary Steps		# Elementary Step Trial (Success rate/%)			
#	Ref.	GFN2	DFTB3	GFN2	DFTB3	GFN2		DFTB3	
**3**	9	41	44	97	102	390	(24.9)	390	(26.2)
**5**	1	7	8	226	229	846	(26.7)	846	(27.1)
**6**	1	2	4	452	215	1855	(24.4)	1855	(11.6)
**8**	2	7	5	39	44	136	(28.7)	136	(32.4)
**9**	1	2	2	8	8	27	(29.6)	27	(29.6)
**10**	6	75	94	6465	6372	28755	(22.5)	28755	(22.2)
**11**	5	24	19	129	77	780	(16.5)	780	(9.9)
**12**	3	18	11	65	50	300	(21.7)	300	(16.7)
**13**	5	21	29	2614	2302	11395	(22.9)	11395	(20.2)
**14**	1	34	24	315	271	1830	(17.2)	1830	(14.8)
**15**	1	11	25	662	438	3662	(18.1)	3662	(12.0)
**18**	1	11	16	163	196	846	(19.3)	846	(23.2)
**19**	1	8	6	42	26	657	(6.4)	657	(4.0)
**20**	1	76	116	17161	14723	92836	(18.5)	92836	(15.9)
**21**	10	27	37	280	152	1830	(15.3)	1758	(8.6)
**22**	1	22	50	1124	954	6669	(16.9)	6669	(14.3)
**23**	1	51	32	757	741	4186	(18.1)	4186	(17.7)
**25**	4	826	1518	33395	30195	305255	(10.9)	305255	(9.9)
**26**	3	137	151	1010	763	5886	(17.2)	5886	(13.0)
**27**	2	585	517	6809	4646	72874	(9.3)	72874	(6.4)
**28**	54	158	147	4552	3380	18491	(24.6)	18491	(18.3)
**29**	1	16	34	1767	1222	6940	(25.5)	6940	(17.6)
**30**	1	96	220	5518	4919	43147	(12.8)	43147	(11.4)
**31**	1	187	394	9727	8565	92836	(10.5)	92836	(9.2)
**32**	1	103	248	15577	13469	89055	(17.5)	89055	(15.1)
**33**	1	103	206	5955	5549	45059	(13.2)	45059	(12.3)
**34**	1	616	1082	73115	53819	487431	(15.0)	487431	(11.0)
**35**	1	53	71	387	298	2211	(17.5)	2211	(13.5)
**36**	1	127	221	1326	986	7381	(18.0)	7381	(13.4)
**37**	1	203	655	73178	51219	434799	(16.8)	434799	(11.8)
**38**	1	155	141	3229	2385	13701	(23.6)	13701	(17.4)
**40**	4	501	463	3646	2512	23855	(15.3)	23855	(10.5)
**41**	1	24	61	128	1976	32896	(0.4)	32896	(6.0)
**42**	5	236	312	701	601	3916	(17.9)	3916	(15.3)

#	# Reactions Ref.	# Reactions GFN2	# Reactions DFTB3	# Elementary Steps GFN2	# Elementary Steps DFTB3	# Elementary Step Trials (Success rate/%) GFN2		# Elementary Step Trials (Success rate/%) DFTB3	
**43**	2	184	160	528	470	5516	(9.6)	5565	(8.4)
**60**	1	0	0	0	0	5	(0.0)	5	(0.0)
**61**	1	0	0	0	0	171	(0.0)	171	(0.0)
**62**	1	12	11	269	140	1898	(14.2)	1898	(7.4)
**63**	1	1	2	3	5	136	(2.2)	136	(3.7)
**64**	1	2	2	17	5	300	(5.7)	300	(1.7)
**65**	1	0	0	0	0	3	(0.0)	3	(0.0)
**66**	1	2	1	8	2	120	(6.7)	120	(1.7)
**67**	1	331	382	17674	12495	128537	(13.8)	128537	(9.7)
**68**	1	106	116	552	607	6551	(8.4)	6551	(9.3)
$\sum$	144	5201	7637	289670	227128	1985970	(14.6)	1985947	(11.4)

References

[1] Zimmerman, P. Reliable Transition State Searches Integrated with the Growing String Method. *J. Chem. Theory Comput.* **2013**, *9*, 3043–3050.

[2] Rasmussen, M. H.; Jensen, J. H. Fast and Automatic Estimation of Transition State Structures Using Tight Binding Quantum Chemical Calculations. *PeerJ Phys. Chem.* **2020**, *2*, e15.

[3] Lavigne, Cyrille and dos Passos Gomes, Gabriel and Pollice, Robert and Aspuru-Guzik, Alan, Automatic Discovery of Chemical Reactions Using Imposed Activation. *ChemRxiv* **2020**, DOI: 10.26434/chemrxiv.13008500.v2.

[4] Grambow, C. A.; Jamal, A.; Li, Y.-P.; Green, W. H.; Zádor, J.; Suleimanov, Y. V. Unimolecular Reaction Pathways of a γ-Ketohydroperoxide from Combined Application of Automated Reaction Discovery Methods. *J. Am. Chem. Soc.* **2018**, *140*, 1035–1048.

[5] Koerstz, M.; Rasmussen, M. H.; Jensen, J. H. Fast and Automated Identification of Reactions with Low Barriers: The Decomposition of 3-Hydroperoxypropanal. *SciPost Chem.* **2021**, *1*, 3.

[6] Guner, V.; Khuong, K. S.; Leach, A. G.; Lee, P. S.; Bartberger, M. D.; Houk, K. N. A Standard Set of Pericyclic Reactions of Hydrocarbons for the Benchmarking of Computational Methods: The Performance of Ab Initio, Density Functional, CASSCF, CASPT2, and CBS-QB3 Methods for the Prediction of Activation Barriers, Reaction Energetics, and Transition State Geometries. *J. Phys. Chem. A* **2003**, *107*, 11445–11459.

[7] Zimmerman, P. M. Single-Ended Transition State Finding with the Growing String Method. *J. Comput. Chem.* **2015**, *36*, 601–611.

[8] Siegbahn, P. E. M.; Crabtree, R. H. Modeling the Solvent Sphere: Mechanism of the Shilov Reaction. *J. Am. Chem. Soc.* **1996**, *118*, 4442–4450.

[9] Leone, A. K.; Souther, K. D.; Vitek, A. K.; LaPointe, A. M.; Coates, G. W.; Zimmerman, P. M.; McNeil, A. J. Mechanistic Insight into Thiophene Catalyst-Transfer Polymerization Mediated by Nickel Diimine Catalysts. *Macromolecules* **2017**, *50*, 9121–9127.

[10] Niu, S.; Hall, M. B. Theoretical Studies of Inorganic and Organometallic Reaction Mechanisms. 15. Catalytic Alkane Dehydrogenation by Iridium(III) Complexes. *J. Am. Chem. Soc.* **1999**, *121*, 3992–3999.

[11] Chan, B.; Gill, P. M. W.; Kimura, M. Assessment of DFT Methods for Transition Metals with the TMC151 Compilation of Data Sets and Comparison with Accuracies for Main-Group Chemistry. *J. Chem. Theory Comput.* **2019**, *15*, 3610–3622.

[12] Goerigk, L.; Hansen, A.; Bauer, C.; Ehrlich, S.; Najibi, A.; Grimme, S. A Look at the Density Functional Theory Zoo with the Advanced GMTKN55 Database for General Main Group Thermochemistry, Kinetics and Noncovalent Interactions. *Phys. Chem. Chem. Phys.* **2017**, *19*, 32184–32215.

[13] Quapp, W.; Kraka, E.; Cremer, D. Finding the Transition State of Quasi-Barrierless Reactions by a Growing String Method for Newton Trajectories: Application to the Dissociation of Methylenecyclopropene and Cyclopropane. *J. Phys. Chem. A* **2007**, *111*, 11287–11293.

[14] Gomer, R.; Kistiakowsky, G. B. The Rate Constant of Ethane Formation from Methyl Radicals. *J. Chem. Phys.* **1951**, *19*, 85–91.

[15] Costentin, C.; Robert, M.; Savéant, J.-M. Activation Barriers in the Homolytic Cleavage of Radicals and Ion Radicals. *J. Am. Chem. Soc.* **2003**, *125*, 105–112.

[16] Cembran, A.; Bernardi, F.; Garavelli, M.; Gagliardi, L.; Orlandi, G. On the Mechanism of the Cis-trans Isomerization in the Lowest Electronic States of Azobenzene: S0, S1, and T1. *J. Am. Chem. Soc.* **2004**, *126*, 3234–3243.

[17] Yamamoto, Y.; Hasegawa, H.; Yamataka, H. Dynamic Path Bifurcation in the Beckmann Reaction: Support from Kinetic Analyses. *J. Org. Chem.* **2011**, *76*, 4652–4660.

[18] Lee, S.; Goodman, J. M. Rapid Route-Finding for Bifurcating Organic Reactions. *J. Am. Chem. Soc.* **2020**, *142*, 9210–9219.

[19] Sobez, J.-G.; Steiner, M.; Reiher, M. qcscine/molassembler: Release 1.2.0. 2022; DOI: 10.5281/zenodo.6695086.

[20] Sobez, J.-G.; Reiher, M. Molassembler: Molecular Graph Construction, Modification, and Conformer Generation for Inorganic and Organic Molecules. *J. Chem. Inf. Model.* **2020**, *60*, 3884–3900.

[21] Mayer, I. Charge, bond order and valence in the AB initio SCF theory. *Chem. Phys. Lett.* **1983**, *97*, 270–274.

[22] Mayer, I. Bond order and valence: Relations to Mulliken's population analysis. *Int. J. Quantum Chem.* **1984**, *26*, 151–154.

[23] Vaucher, A. C.; Reiher, M. Minimum Energy Paths and Transition States by Curve Optimization. *J. Chem. Theory Comput.* **2018**, *14*, 3091–3099.

[24] Brunken, C.; Csizi, K.-S.; Grimmel, S. A.; Gugler, S.; Sobez, J.-G.; Steiner, M.; Türtscher, P. L.; Unsleber, J. P.; Vaucher, A. C.; Weymuth, T.; Reiher, M. qcscine/readuct: Release 4.0.0. **2022**, DOI: 10.5281/zenodo.6695171.

[25] Bensberg, M.; Brunken, C.; Csizi, K.-S.; Grimmel, S. A.; Gugler, S.; Sobez, J.-G.; Steiner, M.; Türtscher, P. L.; Unsleber, J. P.; Weymuth, T.; Reiher, M. qcscine/puffin: Release 1.0.0. **2022**, DOI: 10.5281/zenodo.6695462.
